# The COVID-19 pandemic and social distancing in Nigeria: ignorance or defiance

**DOI:** 10.11604/pamj.supp.2020.35.2.23649

**Published:** 2020-05-28

**Authors:** Ebere Roseann Agusi, Sandra Ifynneke Ijoma, Chizuruoke Stephen Nnochin, Nnaemeka Onyekachi Njoku-Achu, Chika Ignatius Nwosuh, Clement Adebajo Meseko

**Affiliations:** 1 National Veterinary Research Institute, P.M.B 001, Vom, Plateau State, Nigeria; 2 NanoScience Group, Department of Physics and Astronomy, University of Nigeria, Nsukka, Nigeria; 3 Center for Basic Space Science, National Space Research and Development, Abuja, Nigeria

**Keywords:** Coronavirus, pandemic, Nigeria, social distancing, compliance

## Abstract

The severity of the novel 2019 Coronavirus leaves much trepidation, anxiety and desperate measures are taken to curb the pandemic. Such measures according to WHO include hygiene, isolation and social distancing. If clustering of people is considered a major catalyst in the spread of corona virus, social distancing is therefore important for its control. But compliance has remained a concern, especially in Nigeria. We examine the concept and global trends in social distancing in infectious disease control and the negative feedback on public health as revealed in current body of knowledge from news media and other literatures. The risks associated with failure to comply with social distancing as a result of ignorance or defiance are highlighted.

## Commentary

Human survival has often been threatened by diverse plagues since existence. One of such recent threat is an infectious disease, the pandemic of SARS-CoV-2 also known as the Coronavirus disease 2019 (COVID-19). The first case of the novel strain of the Coronavirus was reported in Wuhan, Hubei province in China on November 17, 2019 according to the South Morning China Post [1] and ever since, there has been a world-wide exponential increase in the number of infections and casualties. COVID-19 is an infectious disease that causes respiratory illness with symptoms of cough, fever, and in more severe cases, difficulty in breathing. This disease spreads primarily through contact when an infected person either coughs or sneezes openly, when a person touches a surface or object and then touches the eyes, nose, or mouth. There is currently no standard vaccine or cure for COVID-19, hence, its prevention is strongly recommended. According to the World Health Organization (WHO), preventive measures against this virus includes frequent hand-washing for at least 20 seconds; with soap and running water or using alcohol based hand sanitizer; covering the nose and mouth with disposable tissue or flexed elbow when coughing or sneezing; avoid touching the eyes, nose and mouth if hands are not clean and, avoiding close physical contact (1 meter or 3 feet) also known as social distancing [2]. People who are sick are encouraged to self-isolate to avoid infecting others (if shedding coronavirus) or being infected as the immunity of a sick person is usually compromised. This paper examines current knowledge in literatures and community perception on the impact of socio-distancing on the spread of COVID-19.

The concept of socio-distancing in infectious diseases control: social distancing combined with good respiratory hygiene and hand washing are considered the most feasible way to reduce or delay a pandemic that is on course. The Centers for Disease Control and Prevention (CDC) described social distancing as a set of “methods for reducing frequency and closeness of contact between people in order to decrease the risk of transmission of disease”. In the course of the 2019-2020 coronavirus pandemic, the CDC revised the definition of social distancing as “remaining out of congregate settings, avoiding mass gatherings, and maintaining distance (approximately six feet or two meters) from others when possible”. Therefore, in basic terms, social distancing entails physical distancing. The key reason for physical distancing is to reduce the spread of the virus by contact. WHO elucidates that the instance an infected person coughs or sneezes, droplets containing the virus are deposited on objects and surfaces where people may likely touch and as such anyone in close proximity of about 1-2 meters may be at risk. When appropriate physical distance is maintained, the potential to contract and spread of the virus is reduced [3]. Each country affected by the pandemic has reported similar narratives of social, cultural or religious gatherings where large numbers of people spent extended hours in close proximity promoted the spread of the pandemic. People around the world have not taken physical distancing seriously, and this seems to also be the case in Nigeria. When Italy witnessed its first cases of coronavirus, ‘physical-distancing´ was not one of the measures required by their government, consequently, the population continued with close physical interactions which led to widespread community transmission of the virus.

Global socio-distancing and impact on the spread of COVID-19: in a study to ascertain the cause of the unprecedented global spread of the coronavirus, Hendrick Streeck and colleagues studied the first COVID-19 cluster in Heinsberg, Germany following infection of hundreds of people [4]. They noted that a pattern of singing and dancing that invariably results in rapid spread of the virus. Other studies showed that large crowds played an ominous role in the pandemic´s rapid expansion not only in central Europe, but also in the US and Australia [5]. New York may now be the epicenter of the US outbreak, but the customary revelries around Mardi Gras is believed to have been a catalyst for a large outbreak in New Orleans. At this time, there were no social or physical restrictions during carnival. Only weeks later, on 20 March, the mayor of New Orleans issued a stay-at-home order for the city, with Louisiana ordering statewide restrictions two days later. Howbeit, the city recorded its first death on 13 March, and by 22 March Louisiana had 837 cases, of which about 70% were clustered in New Orleans. COVID-19 also thrives in pious settings. About 2,500 worshippers gathered at the Porte Ouverte Christian church in Mulhouse, in Alsace, France, from 17 to 21 February, for one of the most anticipated annual event in the evangelical calendar [6]. According to Nathalie Schnoebelen, the church´s spokesperson, “during the five days, the worshippers greeted each other, pecked each other on the cheeks, and held hands, sometimes while praying during the services.” It would be almost a month before France went into a national lockdown because of the global trend. However, it was only after the congregation at the Porte Ouverte had dispersed and several worshippers tested positive for the coronavirus that major concerns were raised. By the time it had been identified as a cradle for the virus, church worshippers had unwittingly passed it on to others. A nurse who had been at the Porte Ouverte is alleged to have originated a cluster that further contaminated 250 colleagues at Strasbourg University hospital, where she worked.

In Korea, a 61-year-old woman infected 37 people at her church, in Daegu, South Korea [7]. This woman, called “Patient 31” by Korea’s CDC, developed a fever on 10 February and attended four church services before being diagnosed with COVID-19. Authorities described the outbreak as a “super-spreading event,” as the woman single-handedly transmitted the infection to an unusually high number of people, according to Reuters which subsequently increased confirmed cases in South Korea to 104 with one associated death as at 28 February. Responsively, the South Korean government reacted quickly by issuing a state of emergency. Officials closed schools and public places, shut down sporting events and banned large gatherings. That appeared to have worked as the rate of new known infections in South Korea markedly slowed from a peak of 909 new cases on 29 February to 100 or fewer new cases on most days since mid-March. Convincingly, it is safe to say that cause of all these global outbreaks border on physical distancing, its ignorance or defiance.

Social distancing scenario in Nigeria: the first confirmed incident of the COVID-19 in Nigeria was announced on February 27, 2020, when an Italian citizen arriving Nigeria through the Lagos Airport tested positive for the virus [8]. On March 9, 2020, a second case of the virus was reported in Ogun State, a Nigerian citizen in transit from Milan to Lagos who had contact with the Italian citizen. Afterwards, the Nigerian Health Minister announced that 60 persons who had contact with the index Italian patient were under isolation, 40 persons in Ogun State and 20 in Lagos State. However, there has been an increase in confirmed cases and consequent mortality. As of May 16, 2020, according to report from Nigeria Centre for Disease Control (NCDC), there have been 5621 confirmed cases, 3973 active cases in 34 states including the FCT with 176 deaths and 1472 recoveries [9]. On March, 9, 2020, the Nigerian President in a proactive measure to curtail the spread of this virus declared national border closures, State of emergency in the health sector was ordered and cessation of all movements in the FCT, Lagos State and Ogun State for an initial period of 14 days. Relatedly, other states of the federation taking cue, initiated partial lock-downs with each closing their respective borders. During this period, businesses, markets, religious centers, schools and other public institutions and spaces are to be on temporal shut down. All forms of corporate, social and religious gatherings were prohibited, howbeit; strict adherence to social distancing is expected in exclusive cases. Unfortunately, compliance with the directives has become a challenge as many fail in its adherence either due to ignorance or complete defiance.

Several cases abound where there were partial or zero adherence. For instance, a typical scenario plays out in most public places such as banks where customers seeking to gain access into banking halls clustered outside. In more organized societies, in helping people maintain the required distance apart during this pandemic, standing boxes measuring 2-3 meters are drawn for queue in most public places [10] In states with partial lock-down people are not cognizant of maintaining the required interpersonal distancing. Public buses and taxis are closely packed with passengers as in normal time. Although states were defaulters are subjected to stringent penalties, the degree of compliance is higher. Failure to abide by this breeds danger especially in Nigeria where the quality of our health care systems and our ever increasing population are a bad mix. Could some of these cases of defaulters be attributed to indifference and lackadaisical inclination of most Nigerians or is it a clear case of insubordination and tendency for civil disobedience in developing country where the citizen´s patriotism and respect for government is poor? Such attitude may also be attributable to lack of responsibility by the government to her citizen.

Recent local news that made rounds during the lock down involved celebrities and politicians hosting a house party in the city while under lock down. After being duly arraigned, it was also observed that the caution of physical distancing was also not respected at the court hearing as journalists and onlookers milled together. Another audacious show of defiance to the call for social distancing was on display on a national television, TVC during the funeral of the Chief of Staff to the President. Prior to his death he had tested positive to the COVID-19 virus after a return trip from Germany on March 24, 2020. Despite government´s initial announcement that the burial would be conducted in private to show compliance with the guidelines against the spread of COVID-19, it was observed that a cross section of the sympathizers in attendance were crowded with only very few observing physical distancing. The carelessness did not go without public criticism though the federal government has since apologized. Adherence to the rules and precepts is more important to safeguard lives.

Negative feedback in the enforcement of socio-distancing: in restricting movement and encouraging social-cum-physical distancing, the lock-down having its positives also has its fair share of negative feedback. With cases of civil attacks resulting into deaths of people in the hands of law enforcers. A Nigerian Television Station on April 9, 2020 reported the killing of about six people in Kaduna State by suspected law enforcement agents. Similar case was also reported in Delta State where there was an altercation between the natives and soldiers resulting in the death of a native. Instances of defaulting, defiance and violence bring to question whether the lockdown is really serving its purpose. The apparent psychological state of the people coupled with the harsh socio-economic situation in the country may have contributed to the unrest and resistance. Hence, in the face of provocation and rebellion, law enforcement agents should be humane with civilized sense of duty.

Another critical point of dialogue during a lockdown has to do with insufficient socio-economic cushion for the people especially as it concerns the indigents and the vulnerable in the society. A country where more than 85% of its population survives economically on a day-to-day basis, their means of livelihood is sure to be hampered by lockdown. Considering this reality, challenges of inadequate supplies and meagre palliative measures or none from government raises major concerns. Though the government has reeled out laudable initiatives, very little impact has been made. While some complain about marginalization due to the lopsided delivery of these palliatives, others feel its administration and distribution is grossly politicized and not transparent. More so, in some centers where these distributions are conducted, it is observed that social distancing is usually the last thing on the mind of the beneficiaries a succinct description of the struggle between life and livelihood. In combating this COVID-19 pandemic, the government, civil society organizations and humanitarian agencies can ameliorate the living conditions of the less-privileged by being proactive and deliberate in meeting their basic need so as to prevent needless death caused by poverty and hunger. Though there remain a genuine and exigent need for ‘social distancing´, but what this really calls for is ‘physical distancing´ as people can remain socially active whilst maintaining physical distance. Instead of the usual visit to/by family and friend, the social media affords people the opportunity to connect, stay in touch and be entertained. As imperative as it, it is hard to ask people to abruptly alter their social norms and instinctive habits. However, desperate time calls for desperate measures. Maintaining physical distancing is one of the proven measures to limit the spread of coronavirus, so people must learn to change their lifestyle to stay alive.

## Conclusion

There is an obligation to intentionally stay safe during this pandemic period. This is because the global impact of COVID-19 pandemic, including socio-economic fallout of the scourge, is far-reaching than most can imagine, hence doing the needful in abiding by preventive processes should not be difficult. As it is often said, prevention is better than cure. It is better to stay safe.

## Competing interests

The author declares no competing interests.
